# A Ranking Procedure by Incomplete Pairwise Comparisons Using Information Entropy and Dempster-Shafer Evidence Theory

**DOI:** 10.1155/2014/904596

**Published:** 2014-08-27

**Authors:** Dongbo Pan, Xi Lu, Juan Liu, Yong Deng

**Affiliations:** Faculty of Computer and Information Science, Southwest University, Chongqing 400715, China

## Abstract

Decision-making, as a way to discover the preference of ranking, has been used in various fields. However, owing to the uncertainty in group decision-making, how to rank alternatives by incomplete pairwise comparisons has become an open issue. In this paper, an improved method is proposed for ranking of alternatives by incomplete pairwise comparisons using Dempster-Shafer evidence theory and information entropy. Firstly, taking the probability assignment of the chosen preference into consideration, the comparison of alternatives to each group is addressed. Experiments verified that the information entropy of the data itself can determine the different weight of each group's choices objectively. Numerical examples in group decision-making environments are used to test the effectiveness of the proposed method. Moreover, the divergence of ranking mechanism is analyzed briefly in conclusion section.

## 1. Introduction

The increasing trend toward decision-making in various fields requires computational methods for discovering the preferences of ranking. In fact, methods for finding and predicting preferences in a reasonable way are among the very hot topics in recent science study, such as information systems [1], control systems [2], social choices [3, 4], and so on.

The term “pairwise comparisons” generally refers to any process of comparing entities in pairs to judge which of each entity is preferred or has a greater amount of quantitative property. Prominent psychometrician Thurstone first introduced a scientific approach to use pairwise comparisons for measurement in 1927, which he referred to as the law of comparative judgment [5]. Thurstone demonstrated that the method can be used to order items along a dimension such as preference or importance using an interval-type scale. The Bradley-Terry-Luce (BTL) model was applied to pairwise comparison data to scale preferences [6, 7]. The BTL model was identical to Thurstone's model if the simple logistic function was used. Thurstone used the normal distribution in applications of the model, which the method of pairwise comparisons was used as an approach to measuring perceived intensity of physical stimuli, attitudes, preferences, choices, and values. He also studied implications of the theory he developed for opinion polls and political voting [8]. If an individual or organization expresses a preference between two mutually distinct alternatives, this preference can be expressed as a pairwise comparison. If pairwise comparisons are in fact transitive, then pairwise comparisons for a list alternatives (*A*_1_, *A*_2_, *A*_3_,…, *A*_*n*−1_, *A*_*n*_) can take the form

(1) $$\begin{array}{c} A_{1}⪰A_{2}⪰A_{3}⪰\cdots ⪰A_{n-1}⪰A_{n} \end{array}$$

and it means that the alternative *A*_*i*_ is preferred to *A*_*j*_, in which *i* < *j*. Or the alternatives can be expressed as

(2) $$\begin{array}{c} A_{1}≻A_{2}≻A_{3}≻\cdots ≻A_{n-1}≻A_{n} \end{array}$$

and it means that the alternative *A*_*i*_ is strictly preferred to *A*_*j*_, if *i* < *j*.

Although pairwise comparison is a well-known technique in decision-making, in some cases we have to be faced with the problem of incomplete judgement to preference. For instance, if the number of the alternatives *n* is large, the experts may not give the full comparison one by one. In order to overcome such problem, a decision support system (DSS) based on fuzzy information axiom (FIA) is developed in [9]. However, calculation procedure of information axiom is not only incommodious but also difficult for decision makers and it is hard to deal with the incomplete pairwise problems. For the purpose of reducing the complexity of calculation and preference eliciting process, [10] points out that some comparison between alternative can be skipped and a method is proposed to derive the priorities of *n* alternatives from an incomplete *n* × *n* pairwise comparison matrices in [11]. Furthermore, [12] introduces a fuzzy multiexpert multicriteria decision-making method in possibility measure to handle the difficulty of conflict aggregation process. Moreover, the well-known methods of eigenvector or geometric mean are used to ranking pairwise comparison also introduced in [11, 13]. But all of the above references only focus on the complexity of calculation with the complete comparison. However, some methods have been proposed to solve the incomplete comparison problem. Shiraishi et al. proposed a heuristic method which is based on a property of a coefficient of the characteristic polynomial of pairwise comparison matrices [14]. But the solving is mainly depending on the polynomial which has infinitely many solutions, and it is difficult to get the best candidate. In [15], a least squares type method is proposed to directly calculate the priority vector as the solution of a constrained optimization problem instead of calculating the missing entries of pairwise comparison matrices. In [16], a new centroid-index ranking method of fuzzy number in decision-making was proposed by Yong and Qi. He also proposed a method to concern with the ranking of decision alternatives based on preference judgments made on decision alternatives over a number of criteria to Multiple-criteria decision-making (MCDM) problem in [17]. And in fuzzy group decision-making, an optimal consensus method, in which the limit of each expert's compromise was under consideration in the process of reaching group consensus, was proposed by Liu et al. in [18]. Similarly, in order to calculate the priority vector of incomplete preference, a fuzzy preference relation by a goal programming approach was proposed by Xu in [19], and then he developed a method for incomplete fuzzy preference [20]. Subsequently, the eigenvector method and least square method are proposed with incomplete fuzzy preference relation in [21, 22]. The fuzzy decision-making can not only reduce the complexity of calculation, but also be used in incomplete pairwise comparison, but it is important that the little variety of preference will change the result mainly. Moreover, a method for learning valued preference structures using a natural extension of so-called pairwise classification was proposed by Hullermeier and Furnkranz, which may have a potential application in fuzzy classification [23, 24]. A weighted voting procedure is used in the Hullermeier's proposed method, but the problem is that the voting procedure is always lost in the dead cycle.

In this paper, a discount rate is derived from the information entropy, which determines the certainty or uncertainty of the preference. Then an improved method to calculate the probability assignment of preference using an index introduced in [25] with the discount rate is proposed. Comparing to the current method, the incomplete comparison results of the proposed method are completely dependendent on the information itself instead of the human factor. The paper is organized as follows. Some fundamental and quantifying principles of Dempster-Shafer evidence theory are given in Section 2. Then, the ranking procedures by comparing with single group of experts and with independent groups are introduced in Section 3. In this section, the Dempster-Shafer evidence theory model and imprecise Dirichlet model, which give a basic ranking, are concerned. Next, the proposed enhanced method on the case when comparisons are supplied by independent groups of experts is presented in Section 4. In this section, a weight derived from the entropy of BPA's probability is used in Dempster-Shafer evidence theory and improves the existing ranking method mentioned in the previous section. Finally, the conclusions are given in Section 5.

## 2. Preliminaries

### 2.1. Dempster-Shafer Evidence Theory

Dempster-Shafer evidence theory can be divided into probability distribution function, plausibility function, and Dempster evidence combination rule [26, 27]. It is rather flexible for many applied problems.

**Definition 1 .**

Assume frame of discernment is *θ*; then function *m* : 2^*θ*^ → [0,1] satisfies *m*(*ϕ*) = 0, and ∑_*A*∩*θ*_ *m*(*A*) = 1 is called the basic probability distribution of frame of discernment *θ*.

For ∀*A* ⊂ *θ*, *m*(*A*) is the basic probability of *A*. The meaning of *m*(*A*) is that if *A* ⊂ *Ω* and *A* ≠ *Ω*, thus *m*(*A*) is the accurate trust degree of *A*; if *A* = *Ω*, thus *m*(*A*) means that the trust degree of *A* can not be allocated accurately.

**Definition 2 .**

As for ∀*A* ⊂ *θ*, the defined function Bel : *m* : 2^*θ*^ → [0,1] by Bel(*A*) = ∑_*B*⊂*A*_ *m*(*B*) is called the belief function of *θ*.

**Definition 3 .**

As for ∀*A* ⊂ *θ*, pl is called the plausibility function of Bel in $\text{pl}(A)=1-\text{Bel}(\overset{-}{A})$.

The relation between belief function and plausibility function is that Bel(*A*) and pl(*A*) are, respectively, referred to as the lower limit and the upper limit function of pl(*A*) ≥ Bel(*A*).

Even if they are the same evidences, the probability assignment might be different when they came from different sources. Then the orthogonal method is used to combine these functions by dempster-shafer evidence theory.

Assume *m*_1_, *m*_2_,…, *m*_*n*_ are the basic probability assignment functions of 2^*Ω*^, and their orthogonal *m* = *m*_1_ ⊕ *m*_2_ ⊕ ⋯⊕*m*_*n*_ are

(3) m(ϕ)=0,m(A)=k·∑∩Ai=A ∏1≤i≤nmi(Ai), A≠ϕ

in which *k* ^−1^ = 1 − ∑_∩*A*_*i*_=*A*_∏_1≤*i*≤*n*_ *m*_*i*_(*A*_*i*_).

Several of the algorithms basic to Dempster-Shafer's evidence theory are as follows.

(1) It is known that if we assume frame of discernment of some field is *Ω* = *S*_1_, *S*_2_,…, *S*_*n*_ and propositions *A*, *B*,… are the subsets of *Ω*, the inference rule shall be
  (4) $$\begin{array}{c} \text{If}\;\;E\;\;\text{then}\;\;H,\text{CF} \end{array}$$
  among which *E*, *H* are the logic groupings of the proposition, CF is the certainty factor, which is measured by *c*_*i*_ and called credibility. For any proposition *A*, the certainty factor CF of credibility *A* shall satisfy
  (a) *c*_*i*_ ≥ 0, 1 ≤ *i* ≤ *n*,
  (b) ∑_1≤*i*≤*n*_ *c*_*i*_ ≤ 1.
(2) Evidence description: assume *m* is the defined basic probability assignment function of  2^*Ω*^, then it shall meet the following conditions during calculation:
  (a) *m*({*S*_*i*_}) ≥ 0, *S*_*i*_ ∈ *Ω*,
  (b) ∑_1≤*i*≤*n*_(*m*({*S*_*i*_}) ≥ 0) ≤ 1,
  (c) *m*(*Ω*) = 1 − ∑_1≤*i*≤*n*_ *m*({*S*_*i*_}),
  (d) *m*(*A*) = 0,  *A* ⊂ *Ω*,  and |*A*| > 1 or |*A*| = 0  among which |*A*| means the factor numbers of proposition *A*.
(3) Inaccurate inference model:
  (a) suppose *A* is one part proposition of regular condition. Under the condition of evidence *E*, the matching degree of proposition *A* and evidence *E* is
    (5) $$\begin{array}{c} \text{MD}\left(A,E\right)=\{\begin{array}{cc} 1, & \text{if}\;\;E⊃A,\\ 0, & \text{otherwise}, \end{array} \end{array}$$
  (b) the definition of part proposition *A* in regular condition is
    (6) $$\begin{array}{c} \text{CER}=\text{MD}\left(A,E\right)\cdot f\left(A\right). \end{array}$$

### 2.2. Quantifying the Uncertainty in the Dempster-Shafer Evidence Theory

The uncertain factor considered in evidence theory includes both the uncertainty associated with randomness and the uncertainty associated with granularity. The measure of the granular uncertainty associated with a subset is the specificity measure introduced by [28].

**Definition 4 .**

Let *B* be a nonempty subset of *X*. A measure of specificity of *B*, Sp(*B*), is defined, using Card to denote the cardinality of a set, as

(7) $$\begin{array}{c} \text{Sp}\left(B\right)=1-\frac{\text{Card}\left(B\right)-1}{\text{Card}\left(X\right)-1}=\frac{\text{Card}\left(X\right)-\text{Card}\left(B\right)}{\text{Card}\left(X\right)-1}. \end{array}$$

It essentially measures the degree to which *B* has exactly one element. The larger the specificity, the lesser the uncertainty. In [29], the measure to the case of a probability assignment function *m* was extended.

**Definition 5 .**

Assume *m* has focal element *B*_*j*_, *j* = 1 to *q*. Then

(8) $$\begin{array}{c} \text{Sp}\left(m\right)=\overset{q}{\underset{j=1}{\sum }}\text{Sp}\left(B_{j}\right)m\left(B_{j}\right) \end{array}$$

can be denoted as the expected specificity of the focal elements.

Noting that Sp(*m*)∈[0,1] and the larger the Sp(*m*), the less the uncertainty will be. It is clear that the specificity is smallest when *m* is the vacuous belief function, *m*(*X*) = 1. In this case, Sp(*m*) = 0.

Klir [30] and Ayyub [31] introduced a related measure which he called nonspecificity.

**Definition 6 .**

If *m* is a belief function with focal element *B*_*j*_, *j* = 1 to *q*, then its nonspecificity is defined as

(9) $$\begin{array}{c} N\left[\text{Sp}\left(m\right)\right]=\overset{q}{\underset{j=1}{\sum }}\ln\left(\left|B_{j}\right|\right)m\left(B_{j}\right), \end{array}$$

where |*B*_*j*_| = Card(*B*_*j*_).

It is clear that this takes its largest value ln⁡(*n*), where *n* is the cardinality of *X* for the vacuous belief function. It takes its smallest value when *m* is Bayesian where |*B*_*j*_ | = 1; in this case ln⁡(|*B*_*j*_|) = 0 and *N*[Sp(*m*)] = 0.

Yager [32] made this definition of nonspecificity cointensive with the preceding definition of specificity by normalization and negation; hence

(10) $$\begin{array}{c} \text{S}{\text{p}}_{2}\left(m\right)=1-\frac{N\left[\text{Sp}\left(m\right)\right]}{\ln\left(n\right)},\\ \text{S}{\text{p}}_{2}\left(m\right)=1-\frac{1}{\ln\left(n\right)}\overset{q}{\underset{j=1}{\sum }}\ln\left(\left|B_{j}\right|\right)m\left(B_{j}\right). \end{array}$$

The standard measure of uncertainty associated with a probability distribution is the Shannon entropy.

**Definition 7 .**

If *X* = {*x*_1_,…, *x*_*n*_} and if *P* is a probability distribution on *X* such that *p*_*i*_ is the probability of *x*_*i*_, then the Shannon entropy of *P* is

(11) $$\begin{array}{c} H\left(P\right)=-\overset{q}{\underset{i=1}{\sum }}p_{i}\ln\left(p_{i}\right). \end{array}$$

Any extension of this to belief function must be such that it reduced to the Shannon entropy when the belief structure is Bayesian. Yager [29] suggested an extension of the Shannon entropy to belief structures using the measure of dissonance.

**Definition 8 .**

If *m* has focal element *B*_*j*_, then the extension of the Shannon entropy to belief structures is

(12) $$\begin{array}{c} H\left(m\right)=-\overset{q}{\underset{j=1}{\sum }}\ln\text{Pl}\left(B_{j}\right)m\left(B_{j}\right). \end{array}$$

**Figure 1:**
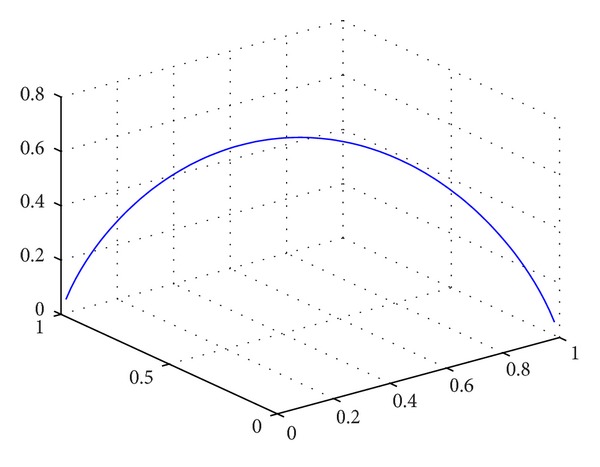
The relationship between the assignment of element and entropy.

For a given set of weights, it can be noted that the smaller the focal elements, the larger the entropy. In particular, the smallest a nonempty set can be is one element. And as we knew, the larger the entropy, the larger the uncertainty. For a given set of weights, the smallest entropy for a Bayesian belief structure shall be one element, because one element always means very certainty information of the preference and the entropy is 0, and the largest entropy occurred when all elements have equal probability. From this, we can conclude that, for any uncertainty and certainty information, the fewer the element the smaller the entropy, and the sparser the probability of element the smaller the entropy.  Figure 1 shows the relationship between the assignment of element and entropy, in which the more balance the assignment of element, the higher the entropy.

## 3. Ranking Procedure by Incomplete Pairwise Comparison

Here we suppose there is a set of alternative Λ = {*A*_1_, *A*_2_,…, *A*_*n*_} with *n* elements. Then we can get 2^*n*^ − 1 subset with *n* elements as

(13) Ω={{A1},…,{An},{A1,A2},…{An−1,An},…,  {A1,…,An}}.

The pairwise comparison means that an expert has chosen some subset of alternatives from *Ω* and compared them pairwisely. If the paired comparisons have been done for all the subset in *Ω*, we call it complete pairwise comparison, otherwise, we call it incomplete pairwise comparison. For complete pairwise comparison, we always get the pairwise comparison matrix that looks like Table 1 in which we give two alternatives as an example. It is supposed that experts only compare subset of alternatives without providing preference value or weights of preference. If an expert chooses one comparison preference, then the value 1 is added to the corresponding cell in the pairwise comparison matrix. For example, *c*_12_ is the number of experts who choose the comparison preference {*A*_1_}⪰{*A*_2_}. There are many references [13, 23, 33] that have studied the complete pairwise comparison matrix. But in most occasions which have a lot of alternatives, the experts can not give the preference one by one. They only choose limited preferences of all the alternatives. Thus, we get the incomplete pairwise comparison matrix that looks like Table 2 with three alternatives, which contain some uncertainty and (or) conflicted information. In complete pairwise comparison matrix, we can calculate the probability of each subset {*A*_*i*_} with *c*_*ij*_, but, in incomplete pairwise comparison, we can not get the assignment of the probability of all the subset. For instant, if an expert chooses the preference {*A*_1_}⪰{*A*_2_, *A*_3_}, we do not know how the probability is distributed among the preferences {*A*_1_}⪰{*A*_2_} and {*A*_1_}⪰{*A*_3_} without any other supplement information. In such situation, we can apply the framework of Dempster-Shafer evidence theory to the considered sets of preferences. Now we suppose there are three alternatives which need to prefer each other. In order to simplify the format, we denote all the subset of the three alternatives as in Table 3, and denote *B*_*ij*_ as the preference *B*_*i*_⪰*B*_*j*_. For example, *B*_16_ means the preference {*A*_1_}⪰{*A*_2_, *A*_3_}. Then we define its BPA for every pairwise comparison in the extended pairwise comparison matrix as follows:

(14) $$\begin{array}{c} m\left(B_{i}⪰B_{j}\right)=m\left(B_{ij}\right)=\frac{c_{ij}}{N},\;\;N=\underset{i,j\in \left\{1,2,\ldots ,n\right\},i\neq j}{\sum }c_{ij}. \end{array}$$

### 3.1. The Ranking Method with One Group of Preference

We assume the experts choose the following preferences from Utkin's works [25]:

 five experts: {*A*_1_, *A*_3_}⪰{*A*_2_} = *B*_52_,
 two experts: {*A*_3_}⪰{*A*_1_, *A*_2_, *A*_3_} = *B*_37_,
 three experts: {*A*_1_}⪰{*A*_3_} = *B*_13_.

Using the Dempster-Shafer evidence theory we described in previous section, we can get the belief function of the preferences {*A*_*i*_}⪰Λ which means {*A*_*i*_} is the best choice of all the subset (or alternative):

 Bel({*A*_1_}⪰Λ) = *m*(*B*_13_) = 0.3,
 Bel({*A*_2_}⪰Λ) = 0,
 Bel({*A*_3_}⪰Λ) = *m*(*B*_37_) = 0.2

and the plausibility functions of the preference:

 pl({*A*_1_}⪰Λ) = *m*(*B*_52_) + *m*(*B*_13_) = 0.8,
 pl({*A*_2_}⪰Λ) = 0,
 pl({*A*_3_}⪰Λ) = *m*(*B*_52_) + *m*(*B*_37_) = 0.7.

From the belief function and the plausibility function, it can be concluded that the best ranking of the alternatives is *A*_1_⪰*A*_3_⪰*A*_2_.

### 3.2. The Ranking Method with Two Independent Groups of Preference

In fact, in order to obtain more consensus result of the preference, we always choose more than one independent group experts to give their preference of alternatives. Thus we can use the well-established method for combining the independent information with the Dempster-Shafer evidence rule of combination.

Here we suppose there are two groups of experts without loss of generality, and denote the preference obtained from the first and second groups of experts by upper indices (1) and (2), respectively. The combined rule refers to the contents introduced in preliminaries. Now we assume that the first group (with five experts) provides the following preferences:

 two experts (*c*_11_ = 2): {*A*_1_}⪰{*A*_2_, *A*_3_} = *B*_16_^(1)^,
 three experts (*c*_12_ = 3): {*A*_1_, *A*_2_}⪰{*A*_3_} = *B*_43_^(1)^.

And the second group (with ten experts) provides the following judgements:

 five experts (*c*_21_ = 5): {*A*_1_, *A*_3_}⪰{*A*_2_} = *B*_52_^(2)^,
 two experts (*c*_22_ = 2): {*A*_3_}⪰{*A*_1_, *A*_2_} = *B*_34_^(2)^,
 three experts (*c*_23_ = 3): {*A*_1_}⪰{*A*_3_} = *B*_13_^(2)^.

Then we get the BPA's of all preferences:

 
*m*_1_(*B*_16_^(1)^) = 0.4,  *m*_1_(*B*_43_^(1)^) = 0.6,
 
*m*_2_(*B*_52_^(2)^) = 0.5,  *m*_2_(*B*_34_^(2)^) = 0.2,  *m*_2_(*B*_13_^(2)^) = 0.3,

and the preference intersections for Dempster-Shafer combination rule are in Table 4.

According to the table, we calculate the weight of conflict first:

(15) K=m1(B16(1))×m2(B34(2))+m1(B43(1))×m2(B52(2)) +m1(B43(1))×m2(B34(2))=0.4×0.2+0.6×0.5+0.6×0.2=0.5.

Then the probability of the assignment for the nonzero combined BPA's preference can be calculated as follows:

(16) m12(B12(12))=11−K×m1(B16(1))×m2(B52(2))=2×0.4×0.5=0.4,m12(B13(12))=11−K×(m1(B16(1))+m1(B43(1)))×m2(B52(2))=2×0.3=0.6.

After that, we can get the belief and plausibility functions of alternatives *A*_1_, *A*_2_, *A*_3_ or preferences {*A*_*i*_}⪰Λ, *i* = 1,2, 3 as follows:

 Bel({*A*_1_}⪰Λ) = *m*_12_(*B*_12_^(12)^) + *m*_12_(*B*_13_^(12)^) = 1,
 pl({*A*_1_}⪰Λ) = 1.

The belief and plausibility functions of {*A*_2_}⪰Λ and {*A*_3_}⪰Λ are 0. So we can only get the result of preference from that the “best” alternative is *A*_1_ and can not get any information of preference about *A*_2_ and *A*_3_. From [25], the author thought the main reason of the situation in previous example was the small number of expert judgements, and the other reason was the used assumption that the sources of evidence were absolutely reliable. So he improved the ranking method with the imprecise Dirichlet model.

### 3.3. The Ranking Method with Imprecise Dirichlet Model

As we described at the final part of the last subsection, the main difficulty of the proposed ranking method is the possible small number of experts. In order to overcome this difficulty, an imprecise Dirichlet model (IDM) introduced by Walley [34] was applied to extend the belief and plausibility functions such that a lack of sufficient statistical data could be taken into account [35, 36]. With the method, we can get the extended belief and plausibility functions as follows:

(17) $$\begin{array}{c} \text{Be}{\text{l}}_{s}\left(A\right)=\frac{N\cdot \text{Bel}\left(A\right)}{N+s},\\ \text{p}{\text{l}}_{s}\left(A\right)=\frac{N\cdot \text{pl}\left(A\right)+s}{N+s}. \end{array}$$

Here the* hyperparameter s* determines how quickly upper and lower probability of events converge as statistical data accumulate, and it should be taken to be 1 or 2; *N* is the number of expert judgements.

However, the main advantage of the IDM is that it produces the cautious inference. In particular, if *N* = 0, then Bel_*s*_(*A*) = 0 and pl_*s*_(*A*) = 1. In the case *N* → *∞*, it can be stated for any *s*: Bel_*s*_(*A*) = Bel(*A*), pl_*s*_(*A*) = pl(*A*). If we denote *μ* = *N*/(*N* + *s*), then there holds m*(*A*_*i*_) = *μ* · *m*(*A*_*i*_). One can see from the last expression for m*(*A*_*i*_) that *μ* is the discount rate characterizing the reliability of a source of evidence and it depends on the number of estimates *N*. Because the total probability assignment ∑*m*(*A*_*i*_) = 1, we assign the left probability to m*(*B*_77_); that means that the experts do not know which alternative is better than the others and is indicated by the preference {*A*_1_, *A*_2_, *A*_3_}⪰{*A*_1_, *A*_2_, *A*_3_}. By using the discount rate *μ* with *s* = 1, we can get *μ*_1_ = 5/6≃0.83 for the first group and *μ*_2_ = 10/11≃0.91. Hence we can rewrite the preference intersection for Dempster-Shafer combination rule in Table 5.

And the modified probability assignment and conflict weight are

(18) $$\begin{array}{c} m_{1}^{*}\left(B_{16}^{\left(1\right)}\right)=0.33,\;\;m_{1}^{*}\left(B_{43}^{\left(1\right)}\right)=0.5,\\ m_{1}^{*}\left(B_{77}^{\left(1\right)}\right)=0.17,\;\;m_{2}^{*}\left(B_{52}^{\left(2\right)}\right)=0.46,\\ m_{2}^{*}\left(B_{34}^{\left(2\right)}\right)=0.18,\;\;m_{2}^{*}\left(B_{13}^{\left(2\right)}\right)=0.27,\\ m_{2}^{*}\left(B_{77}^{\left(2\right)}\right)=0.09,\\ K^{*}=\mu_{1}\mu_{2}K=0.83\cdot 0.91\cdot 0.5=0.378. \end{array}$$

Now the combined BPA in Table 5 are

(19) m12(B12(12))=11−K∗·m1∗(B16(1))·m2∗(B52(2))=1.61·0.33·0.46=0.244,m12(B13(12))=11−K∗·(m1∗(B16(1))+m1∗(B43(1))+m1∗(B77(1))) ·m2∗(B13(2))=1.61·0.27=0.435.

Similarly,

(20)m12(B16(12))=0.048,  m12(B43(12))=0.072,m12(B52(12))=0.126,  m12(B34(12))=0.049,(21)m12(B77(12))=0.026.

So the belief and plausibility functions of {*A*_*i*_}⪰Λ are

(22) Bel({A1}⪰Λ)=m12(B12(12))+m12(B13(12))+m12(B16(12))=0.727,pl({A1}⪰Λ)=Bel({A1}⪰Λ)+m12(B52(12)) +m12(B43(12))+m12(B77(12))=0.951.

In the same way,

(23) $$\begin{array}{c} \text{Bel}\left(\left\{A_{2}\right\}⪰\Lambda \right)=0,\\ \text{pl}\left(\left\{A_{2}\right\}⪰\Lambda \right)=0.098,\\ \text{Bel}\left(\left\{A_{3}\right\}⪰\Lambda \right)=0.049,\\ \text{pl}\left(\left\{A_{3}\right\}⪰\Lambda \right)=0.201. \end{array}$$

It can be seen from the above results that the “best” ranking is *A*_1_⪰*A*_3_⪰*A*_2_.

## 4. Improved Method and Numerical Analysis

The main advantage of IDM method is that it allows us to deal with comparisons of arbitrary groups of alternatives. It gives the possibility to use the framework of Dempster-Shafer evidence theory and to compute the belief and plausibility functions of alternatives or ranking and provides a way to make cautious decisions when the number of expert estimates is rather small. However, the method depends excessively on the number of experts rather than data itself. If we change the number of experts, it may lead to a different result. For instant, we assume the number of first group of experts is 50, and 20 experts choose the preference {*A*_1_}⪰{*A*_2_, *A*_3_} = *B*_16_^(1)^, and 30 experts choose the preference {*A*_1_, *A*_2_}⪰{*A*_3_} = *B*_43_^(1)^. Then we get the same probability assignment for the first group of experts. The only change is the discount rate from 0.83 to *μ*_1_ = 50/51 = 0.98. Here we recompute the probability assignment and conflict weight as follows:

(24) m1∗(B16(1))=0.4·0.98=0.392,m1∗(B43(1))=0.6·0.98=0.588,  m1∗(B77(1))=0.02,m2∗(B52(2))=0.46,  m2∗(B34(2))=0.18,m2∗(B13(2))=0.27,  m2∗(B77(2))=0.09,K∗=μ1μ2K=0.98·0.91·0.5=0.446.

Now we recompute the combined BPA in Table 5

(25) m12(B12(12))=11−K∗·m1∗(B16(1))·m2∗(B52(2))=1.81·0.392·0.46=0.326,m12(B13(12))=11−K∗·(m1∗(B16(1))+m1∗(B43(1))+m1∗(B77(1))) ·m2∗(B13(2))=1.81·0.27=0.489.

Similarly,

(26)m12(B16(12))=0.064,  m12(B43(12))=0.096,m12(B52(12))=0.017,  m12(B34(12))=0.007,(27)m12(B77(12))=0.003.

So the belief and plausibility functions of {*A*_*i*_}⪰Λ are

(28) Bel({A1}⪰Λ)=m12(B12(12))+m12(B13(12))+m12(B16(12))=0.879,pl({A1}⪰Λ)=Bel({A1}⪰Λ)+m12(B52(12)) +m12(B43(12))+m12(B77(12))=0.995.

In the same way,

(29) $$\begin{array}{c} \text{Bel}\left(\left\{A_{2}\right\}⪰\Lambda \right)=0,\\ \text{pl}\left(\left\{A_{2}\right\}⪰\Lambda \right)=0.099,\\ \text{Bel}\left(\left\{A_{3}\right\}⪰\Lambda \right)=0.007,\\ \text{pl}\left(\left\{A_{3}\right\}⪰\Lambda \right)=0.027. \end{array}$$

Thus we get that the “best” ranking is *A*_1_⪰*A*_3_⪰*A*_2_ by pessimistic decision-making and *A*_1_⪰*A*_2_⪰*A*_3_ by optimistic decision-making. Comparing with the result in previous subsection, we will find that we only change the number of experts and then lead to a conflicting result. We think the problem is that the discount rate depending on the number of experts is a subjective parameter. It can not reflect the objectivity of the probability assignment itself. So we obtain different result with the same probability assignment of the incomplete pairwise comparison.

In order to overcome the problem, we introduce an improved method to obtain the discount rate by using entropy which we describe in the Preliminaries section. Because the entropy can measure the uncertainty of the data, it can be applied to extend belief and plausibility function by indicating the sparser of the probability assignment and the fewer of the subset between different groups of pairwise comparison. In particular, if the preference has the probability assignment function *m* with focal element *B*_*ij*_, we define the entropy of the preference as

(30) $$\begin{array}{c} p\left(m\right)=-\overset{q}{\underset{i,j=1}{\sum }}\ln\left(m\left(B_{ij}\right)\right)m\left(B_{ij}\right). \end{array}$$

**Figure 2:**
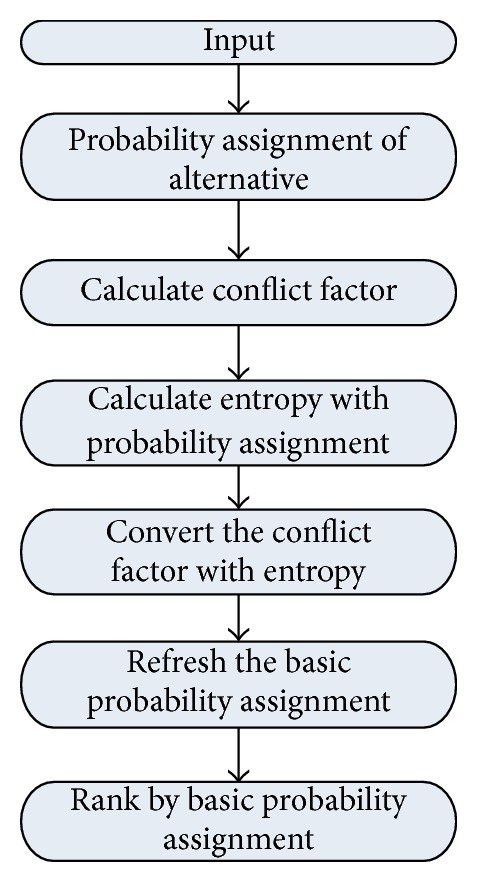
The flowchart of the improved method.

Among the definition, *q* = 2^*n*^ − 1, and *n* is the number of alternatives. Each group of experts can give their preference of alternatives with pairwise comparison, and then we can calculate every entropy of each group. Because the more uncertain the probability assignment function, the greater the entropy, and the lesser the discount rate of preference assigned by the group of experts; we denote *x* = 1/*p*(*m*) as the discount rate. After that, we get a sequence of the discount rate. The sequence of the discount rate can be normalized by the formula *y* = *a* tan(*x*) · 2/*π*, *x* is the input data before normalization which is equal to the discount rate, and *y* is the output data after normalization. Noting that the dimension of *p*(*m*) is the *m* ^2^(*B*_*ij*_), we define the weight as $\mu =\sqrt{y}$. Furthermore, we get the weight of each group of probability assignment in the incomplete pairwise comparison matrix. Without loss of generality, if *m*(*B*_*ij*_) = 0, we define ln⁡(*m*(*B*_*ij*_))*m*(*B*_*ij*_) = 0. The flowchart of the improved method using entropy to convert the conflict factor is following in Figure 2.

Now we recalculate the previous example as follows:

(31) p1(m)=−∑i,j=1qln⁡(m(Bij))m(Bij)=−(0.4 ln⁡0.4+0.6 ln⁡0.6)=0.673,p2(m)=−∑i,j=1qln⁡(m(Bij))m(Bij)=−(0.5 ln⁡0.5+0.2 ln⁡0.2+0.3 ln⁡0.3)=1.0297.

Then we get the discount rate for two groups:

(32) $$\begin{array}{c} x_{1}=1.4859,\;\;x_{2}=0.9712. \end{array}$$

Next we compute the normalized discount rate:

(33) $$\begin{array}{c} y_{1}=0.6229,\;\;y_{2}=0.4907. \end{array}$$

Last we obtain the weights of each group:

(34) $$\begin{array}{c} \mu_{1}=0.79,\;\;\mu_{2}=0.7. \end{array}$$

Then the next thing is to recompute the probability assignment and conflict weight as follows:

(35) m1∗(B16(1))=0.4·0.79=0.316,m1∗(B43(1))=0.6·0.79=0.474,  m1∗(B77(1))=0.21,m2∗(B52(2))=0.5·0.7=0.35,  m2∗(B34(2))=0.2·0.7=0.14,m2∗(B13(2))=0.3·0.7=0.21,  m2∗(B77(2))=0.3,K∗=μ1μ2K=0.79·0.7·0.5=0.2765.

Now we recompute the combined BPA in Table 5:

(36) m12(B12(12))=11−K∗·m1∗(B16(1))·m2∗(B52(2))=1.3822·0.316·0.35=0.1529,m12(B13(12))=11−K∗·(m1∗(B16(1))+m1∗(B43(1))+m1∗(B77(1))) ·m2∗(B13(2))=1.3822·0.21=0.0803.

Similarly,

(37)m12(B16(12))=0.131,  m12(B43(12))=0.1965,m12(B52(12))=0.1016,  m12(B34(12))=0.0406,(38)m12(B77(12))=0.0871.

So the belief and plausibility functions of {*A*_*i*_}⪰Λ are

(39) Bel({A1}⪰Λ)=m12(B12(12))+m12(B13(12))+m12(B16(12))=0.4297,pl({A1}⪰Λ)=Bel({A1}⪰Λ)+m12(B52(12)) +m12(B43(12))+m12(B77(12))=0.8149.

In the same way, we can get other belief and plausibility functions:

(40) $$\begin{array}{c} \text{Bel}\left(\left\{A_{2}\right\}⪰\Lambda \right)=0,\\ \text{pl}\left(\left\{A_{2}\right\}⪰\Lambda \right)=0.2836,\\ \text{Bel}\left(\left\{A_{3}\right\}⪰\Lambda \right)=0.0406,\\ \text{pl}\left(\left\{A_{3}\right\}⪰\Lambda \right)=0.2293. \end{array}$$

According to the result of pairwise comparison, we can conclude that the “best” ranking is *A*_1_⪰*A*_3_⪰*A*_2_ by pessimistic decision-making and *A*_1_⪰*A*_2_⪰*A*_3_ by optimistic decision-making. Because the method is not dependent on the amount of the experts, the result will not change with the increasing of experts.

Moreover, we extend the problem to lower or higher conflict with the alternative of experts. Here we assume two different statuses with the alternative of experts, one is lower conflict of alternative and the other is higher conflict of alternative. Then we evaluate the results, respectively.

For lower conflict condition, we assume the first group (with five experts) provides the following preferences:

 two experts (*c*_11_ = 2): {*A*_1_}⪰{*A*_2_, *A*_3_} = *B*_16_^(1)^,
 three experts (*c*_12_ = 3): {*A*_1_, *A*_2_}⪰{*A*_3_} = *B*_43_^(1)^.

And the second group (with ten experts) provides the following judgements:

 five experts (*c*_21_ = 5): {*A*_1_, *A*_3_}⪰{*A*_2_} = *B*_52_^(2)^,
 two experts (*c*_22_ = 2): {*A*_2_}⪰{*A*_3_} = *B*_23_^(2)^,
 three experts (*c*_23_ = 3): {*A*_1_}⪰{*A*_3_} = *B*_13_^(2)^.

Then we get the BPA of all preference:

 
*m*_1_(*B*_16_^(1)^) = 0.4, *m*_1_(*B*_43_^(1)^) = 0.6,
 
*m*_2_(*B*_52_^(2)^) = 0.5, *m*_2_(*B*_23_^(2)^) = 0.2, *m*_2_(*B*_13_^(2)^) = 0.3,

and the preference intersections for lower conflict are in Table 6.

The conflict factors are 0.287 for IDM method and 0.2101 for improved method, respectively, and the rankings are *A*_1_⪰*A*_2_⪰*A*_3_ both by pessimistic decision-making and optimistic decision-making either in IDM method or in improved method.

If we increase the number of experts in first group to 50, 20 experts choose {*A*_1_}⪰{*A*_2_, *A*_3_} and the left experts choose {*A*_1_, *A*_2_}⪰{*A*_3_}. The conflict factor shifts to 0.3091 for IDM method and keeps the same as 0.2101 for improved method, and the ranking is the same as the prior one. The result shows that the IDM method and improved method can maintain the consistency in lower conflict of alternative.

For higher conflict condition, we assume the first group (with five experts) provide the following preferences:

 two experts (*c*_11_ = 2): {*A*_1_}⪰{*A*_2_, *A*_3_} = *B*_16_^(1)^,
 three experts (*c*_12_ = 3): {*A*_1_, *A*_2_}⪰{*A*_3_} = *B*_43_^(1)^.

And the second group (with ten experts) provide the following option:

 five experts (*c*_21_ = 5): {*A*_1_, *A*_3_}⪰{*A*_2_} = *B*_52_^(2)^,
 two experts (*c*_22_ = 2): {*A*_3_}⪰{*A*_1_, *A*_2_} = *B*_34_^(2)^,
 three experts (*c*_23_ = 3): {*A*_2_}⪰{*A*_1_} = *B*_21_^(2)^.

Then we get the BPA of all preferences:

 
*m*_1_(*B*_16_^(1)^) = 0.4, *m*_1_(*B*_43_^(1)^) = 0.6,
 
*m*_2_(*B*_52_^(2)^) = 0.5, *m*_2_(*B*_34_^(2)^) = 0.2, *m*_2_(*B*_21_^(2)^) = 0.3,

and the preference intersections for higher conflict are in Table 7.

The conflict factors are 0.7134 for IDM method and 0.4424 for improved method, respectively, and the rankings are *A*_1_⪰*A*_2_⪰*A*_3_ both by pessimistic decision-making and optimistic decision-making either in IDM method or in improved method.

In the same way, we increase the number of experts in first group to 50, and 20 experts choose {*A*_1_}⪰{*A*_2_, *A*_3_} and 30 experts choose {*A*_1_, *A*_2_}⪰{*A*_3_}. The conflict factor shifts to 0.6042 for IDM method and the same as 0.4424 for improved method. The ranking is *A*_1_⪰*A*_2_⪰*A*_3_ by pessimistic decision-making and *A*_1_⪰*A*_3_⪰*A*_2_ by optimistic decision-making in IDM method and the same result in improved method as prior one. The result indicates that the IDM method can not keep the availability in higher conflict situation and the improved method maintains the effectiveness and reliability both in lower and higher conflict situations. The results of comparison are in Table 8.

Analyzing the result of improved method previous section, there is no doubt that the “best” alternative is {*A*_1_}, and the problem is the ranking between {*A*_2_} and {*A*_3_}. Moreover, we find that 60% of experts of the first group choose the preferences {*A*_1_, *A*_2_}⪰{*A*_3_} and 70% of experts of the second group choose the preferences {*A*_1_, *A*_3_}⪰{*A*_2_} and {*A*_3_}⪰{*A*_1_, *A*_2_} which include the information about {*A*_2_} and {*A*_3_}. So the ranking between {*A*_2_} and {*A*_3_} depends on the weight of the ranking chosen by the first group and the second group of experts. Noticing that the weight of imprecise Dirichlet method relies heavily on the number of experts, the weight of the second group with ten experts is greater than the first group with five experts. Thus the result is propitious to the preferences {*A*_1_, *A*_3_}⪰{*A*_2_} and {*A*_3_}⪰{*A*_1_, *A*_2_}.

## 5. Conclusion

Different from the imprecise Dirichlet method, we propose an improved method to deal with the incomplete pairwise comparison by any groups of alternatives and experts. The proposed method assigns the probability to the belief and (or) plausibility of alternatives or ranking using the weighted DEMPSTER-SHAFER evidence method. Moreover, in order to objectively consider the fairness of decision-making between different groups of experts, the proposed method introduces the entropy, which indicates the value of information, to calculate the weight. The weight in the proposed method is decided by the initial data or probability assignment itself rather than the number of experts. It takes into account the factor of the number of the elements and the sparse degree of the probability assignment and pays more attention on the establishment mechanism of the data. At last, a numerical analysis illustrates the method and shows the difference from the imprecise Dirichlet method.

## Figures and Tables

**Table 1:** The complete pairwise comparison matrix.

	{*A*_1_}	{*A*_2_}	{*A*_1_, *A*_2_}
{*A*_1_}	—	*c*_12_	*c*_13_
{*A*_2_}	*c*_21_	—	*c*_23_
{*A*_1_, *A*_2_}	*c*_31_	*c*_32_	—

**Table 2:** The incomplete pairwise comparison matrix.

	{*A*_1_}	{*A*_2_}	{*A*_3_}	{*A*_1_, *A*_2_}	{*A*_1_, *A*_3_}	{*A*_2_, *A*_3_}	{*A*_1_, *A*_2_, *A*_3_}
{*A*_1_}	—	*c*_12_	—	—	—	*c*_16_	—
{*A*_2_}	*c*_21_	—	—	—	*c*_25_	*c*_26_	—
{*A*_3_}	—	*c*_32_	—	—	—	—	*c*_37_
{*A*_1_, *A*_2_}	—	—	*c*_43_	—	—	—	—
{*A*_1_, *A*_3_}	*c*_51_	*c*_52_	—	—	—	*c*_56_	—
{*A*_2_, *A*_3_}	*c*_61_	—	—	—	—	—	—
{*A*_1_, *A*_2_, *A*_3_}	—	—	—	—	—	—	—

**Table 3:** The subset of three alternatives and their short notations.

{*A*_1_}	{*A*_2_}	{*A*_3_}	{*A*_1_, *A*_2_}	{*A*_1_, *A*_3_}	{*A*_2_, *A*_3_}	{*A*_1_, *A*_2_, *A*_3_}
{*B*_1_}	{*B*_2_}	{*B*_3_}	{*B*_4_}	{*B*_5_}	{*B*_6_}	{*B*_7_}

**Table 4:** The preference intersections for Dempster-Shafer combination rule.

			The first group	
			{*A*_1_}⪰{*A*_2_, *A*_3_}	{*A*_1_, *A*_2_}⪰{*A*_3_}
			*B*_16_^(1)^	*B*_43_^(1)^
The second group	{*A*_1_, *A*_3_}⪰{*A*_2_}	*B*_52_^(2)^	{*A*_1_}⪰{*A*_2_}	*ϕ*
	{*A*_3_}⪰{*A*_1_, *A*_2_}	*B*_34_^(2)^	*ϕ*	*ϕ*
	{*A*_1_}⪰{*A*_3_}	*B*_13_^(2)^	{*A*_1_}⪰{*A*_3_}	{*A*_1_}⪰{*A*_3_}

**Table 5:** The preference intersections for modified Dempster-Shafer combination rule.

			The first group	
		*B*_16_^(1)^	*B*_43_^(1)^	*B*_77_^(1)^
The second group	*B*_52_^(2)^	{*A*_1_}⪰{*A*_2_}	*ϕ*	{*A*_1_, *A*_3_}⪰{*A*_2_}
	*B*_34_^(2)^	*ϕ*	*ϕ*	{*A*_3_}⪰{*A*_1_, *A*_2_}
	*B*_13_^(2)^	{*A*_1_}⪰{*A*_3_}	{*A*_1_}⪰{*A*_3_}	{*A*_1_}⪰{*A*_3_}
	*B*_77_^(2)^	{*A*_1_}⪰{*A*_2_, *A*_3_}	{*A*_1_, *A*_2_}⪰{*A*_3_}	Λ⪰Λ

**Table 6:** The preference intersections for lower conflict.

			The first group	
		*B*_16_^(1)^	*B*_43_^(1)^	*B*_77_^(1)^
The second group	*B*_52_^(2)^	{*A*_1_}⪰{*A*_2_}	*ϕ*	{*A*_1_, *A*_3_}⪰{*A*_2_}
	*B*_23_^(2)^	*ϕ*	{*A*_2_}⪰{*A*_3_}	{*A*_2_}⪰{*A*_3_}
	*B*_13_^(2)^	{*A*_1_}⪰{*A*_3_}	{*A*_1_}⪰{*A*_3_}	{*A*_1_}⪰{*A*_3_}
	*B*_77_^(2)^	{*A*_1_}⪰{*A*_2_, *A*_3_}	{*A*_1_, *A*_2_}⪰{*A*_3_}	Λ⪰Λ

**Table 7:** The preference intersections for higher conflict.

			The first group	
		*B*_16_^(1)^	*B*_43_^(1)^	*B*_77_^(1)^
The second group	*B*_52_^(2)^	{*A*_1_}⪰{*A*_2_}	*ϕ*	{*A*_1_, *A*_3_}⪰{*A*_2_}
	*B*_23_^(2)^	*ϕ*	*ϕ*	{*A*_3_}⪰{*A*_1_, *A*_2_}
	*B*_13_^(2)^	*ϕ*	*ϕ*	{*A*_2_}⪰{*A*_1_}
	*B*_77_^(2)^	{*A*_1_}⪰{*A*_2_, *A*_3_}	{*A*_1_, *A*_2_}⪰{*A*_3_}	Λ⪰Λ

**Table 8:** Results of comparison between Utkin's method and proposed method.

		Results	
		Utkin's method	Proposed method
5 experts	Low conflict factor	{*A*_1_}⪰{*A*_2_}⪰{*A*_3_}	{*A*_1_}⪰{*A*_2_}⪰{*A*_3_}
	High conflict factor	{*A*_1_}⪰{*A*_2_}⪰{*A*_3_}	{*A*_1_}⪰{*A*_2_}⪰{*A*_3_}

50 experts	Low conflict factor	{*A*_1_}⪰{*A*_2_}⪰{*A*_3_}	{*A*_1_}⪰{*A*_2_}⪰{*A*_3_}
	High conflict factor	{*A*_1_}⪰{*A*_2_}⪰{*A*_3_} (pessimistic)	{*A*_1_}⪰{*A*_2_}⪰{*A*_3_}
		{*A*_1_}⪰{*A*_3_}⪰{*A*_2_} (optimistic)	
